# Antitumor effect of isoquercetin on tissue vasohibin expression and colon cancer vasculature

**DOI:** 10.18632/oncotarget.28181

**Published:** 2022-02-08

**Authors:** Daniel de Castilho da Silva, Guilherme Di Camillo Orfali, Maycon Giovani Santana, Jessica Kaoru Yamamoto Palma, Isabella Ramos de Oliveira Assunção, Isadora Moraes Marchesi, Ana Yoshie Kitagawa Grizotto, Natália Peres Martinez, Simone Felliti, José Aires Pereira, Denise Gonçalves Priolli

**Affiliations:** ^1^Programme Stricto Sensu in Health Science, Sao Francisco University Medical School, Sao Paulo, Brazil; ^2^Escola Paulista de Medicina, São Paulo, Brazil; ^3^Scientific Initiation Programme, Sao Francisco University Medical School, Sao Paulo, Brazil; ^4^Department of Oncology, Sao Francisco University Medical School, Sao Paulo, Brazil; ^5^Department of Pathology, Sao Francisco University Medical School, Sao Paulo, Brazil; ^6^Postgraduate Programme Stricto Sensu in Health Science, Sao Francisco University Medical School, Sao Paulo, Brazil; ^*^These authors contributed equally to this work

**Keywords:** target, angiogenic proteins, neoplasms, flavonols, antitumor assays-xenograft model

## Abstract

Tumor cells trigger angiogenesis through the expression of angiogenic factors. Vasohibins (VASHs) are a family of peptides that regulate angiogenesis. Flavonoids have antiproliferative antitumor properties; however, few studies have highlighted their antiangiogenic potential. This study evaluated the flavonoid isoquercetin (Q3G) as an antitumor compound related to colon cancer vascularization and regulation of VASH1 and 2. Mice bearing xenogeneic colon cancer (*n* = 15) were divided into 3 groups: Q3G-treated (gavage, daily over a week), bevacizumab-treated (intraperitoneal, single dose), or untreated animals. Tumor growth, histological characteristics, blood vessel volume, and VASH1 and 2 expressions were analyzed. Q3G impaired tumor growth and vascularization, upregulated VASH1, and downregulated VASH2 in comparison to untreated animals. Mice treated with Q3G showed approximately 65% fewer blood vessels than untreated animals and 50% fewer blood vessels than mice treated with bevacizumab. Thus, we show that Q3G has antitumor activity, impairs vascularization, and differentially modulates VASH1 and 2 expressions in colon cancer.

## INTRODUCTION

Angiogenesis is characterized by the establishment of new blood vessels (BV) through stimulation of endothelial proliferation. The steps involved in angiogenesis include endothelial cell (EC) proliferation, sprouting, migration, tube formation, vessel remodeling, and pruning. In healthy tissues, angiogenesis is regulated by natural signals that prevent or stimulate the overgrowth of neovascularization [1].

Angiogenesis is a complex and multifactorial process that includes, but is not limited to, (i) stimulation by pro-angiogenic factors such as vascular endothelial growth factor (VEGF), basic fibroblast growth factor (bFGF), and transforming growth factor-beta (TGF-β); (ii) repression by anti-angiogenic factors (angiostatin, endostatin, and vasoinhibin); and (iii) regulation by non-angiogenic factors (O_2_ consumption rate and nutrient deprivation threshold for early necrosis) [2–4]. Fibroblast growth factors (FGF) and VEGF-induced signal transduction led to specific biological responses. Both, (FGFs) and VEGFs, stimulate endothelial cells to secrete several proteases such as vascular endothelial growth factor receptor 2 (VEGFR2) and fibroblast growth factor receptor 1 (FGFR1). Thereby, signaling mediated by angiogenic stimulators, modulate the vasohibin (VASH) protein family. VASH1 and 2 are peptides linked to opposing angiogenic regulation processes [5]. VASH1 is an anti-angiogenic factor. It increases tubulin levels and thereby suppresses endocytosis, whereas VASH2 is pro-angiogenic and exhibits tubulin carboxypeptidase activity related to microtubule functions and facilitates tubulin detyrosination. Small vasohibin-binding protein (SVBP) is a protein that regulates the abundance of VASH1 and 2. VASHs, but not SVBP alone, increase detyrosination of α-tubulin, and purified vasohibins remove the C-terminal tyrosine of α-tubulin [6]. The activities of VASH1 and 2 activities are linked to their expression sites. VASH1 is mainly produced by ECs [7], and VASH2 is mainly derived from infiltrating mononuclear cells [8]. VASH2 has also been detected in tumor cells [9]. VASH1 levels are low in proliferating ECs at the sprouting front and high in non-proliferating ECs at the angiogenesis termination zone where VASH1 possibly interrupts angiogenesis. Conversely, high levels of VASH2 are found at the sprouting front and lower levels at the termination zone [9].

Dysregulation of angiogenesis contributes to the development of numerous diseases, including cancer [2, 3, 5, 10–14]. Cancer is “angiogenesis-dependent” [15] because it depends on neovasculature to fulfill the metabolic demands of cancer cell proliferation. Tumor vasculature offers an excellent and potentially selective target for anticancer therapy. Thus, significant advances in cancer treatment have been achieved with the development of antiangiogenic agents [16].

The overarching realm of vascular targeting strategies include both angiogenesis inhibiting agents (AIAs) and vascular disrupting agents (VDAs), which are collectively described as vascular targeting agents (VTAs)[17–21].

AIAs prevent the formation of new BV without acting on the pre-formed BV, and this limits tumor growth by blood deprivation [22]. Administration of AIAs can result in tumor cell necrosis and secondary tumor cell death [17–19, 21]. Bevacizumab (beva) is classified as an AIA [23]. Beva is an anti-VEGFR antibody. It is a recombinant humanized monoclonal immunoglobulin G1 (IgG1), that contains a human framework region and a murine complementarity-determining region. It targets the VEGF receptor signaling pathway and blocks angiogenesis through ligand binding and sequestration. Beva causes EC apoptosis by blocking VEGFR1 [24], thus impairing cell proliferation, migration, survival, and vascular permeability [23]. Beva has been approved for several cancer indications, including breast cancer [25]; first-line non-squamous non-small cell lung cancer [26]; recurrent glioblastoma [27]; metastatic renal cell carcinoma [28]; persistent, recurrent, or metastatic cervical cancer [29]; epithelial ovarian cancer [30]; fallopian tube cancer; and primary peritoneal cancer [31]. Beva is the second-line treatment for patients with metastatic colon cancer (CC) who have progressed on a first-line beva-containing regimen [18, 19]. Unfortunately, beva, aflibercept, and ramucirumab, like many other drugs, such as unitinib and pazopanib, that are designed to act specifically on a target proteins or receptors [32], may bind to unintended proteins and can exhibit off-target activity and adverse effects [18, 19, 21, 25].

VDAs are an emerging class of anticancer agents. They damage and destroy the existing tumor vasculature. VDAs depolymerize tubulin to disrupt tumor vascularization [20]. One subset of VDA functions is inhibition of microtubular tubulin, which leads to morphological changes to the ECs lining the tumor vasculature and triggers a cascade of cell signaling events that result in BV damage. Flavonoids, such as flavone-8-acetic acid (FAA) [33] and dimethylxanthenone-4-acetic acid (DMXAA) or vadimezan are VDAs [34]. Another flavonoid, kaempferol, protects against retinal damage by regulating VASH1 and destroys retinal neovasculature [35]. Thus, the literature suggests that flavonoids are also a group of VDAs [33, 34].

Flavonoids have been shown to have effects on ovarian [36], breast [37, 38], prostate [39], liver [40], and colorectal cancer [41, 42]. Flavonoids are characterized by a phenyl benzo(y)pyrone-derived structure with putative anticancer effects [43–48]. While clinical use of quercetin is limited by its poor bioavailability, quercetin-3-O-β-d-glucopyranoside (Q3G or isoquercetin) has a better pharmacokinetic profile than quercetin [43–51]. It has an antiproliferative effect *in vitro* on CC cells (SW480, DLD-1, and HCT116) no significant effect on non-tumor colon cells (IEC-18) [52]. Considering this antiproliferative effect of Q3G on CC [52, 53], the evidence from flavonols as promising angiogenic agents, and also its relationship with VASHs [35, 54], we evaluated the antitumoral effect of Q3G *in vivo* and its relationship with vascularization in a xenogeneic CC animal model.

## RESULTS

### CC animal model features

**Figure 1:**
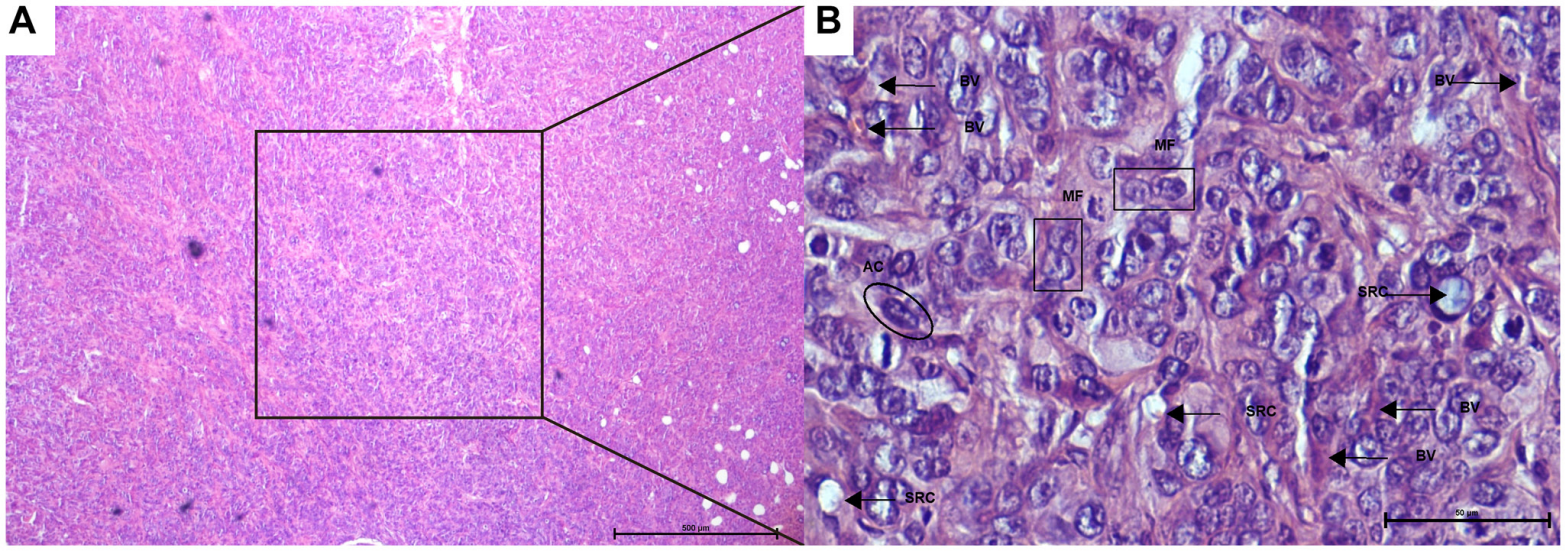
Photomicrographs of colon cancer histopathological features (**A**, **B**) following xenogeneic HT-29/CC cells seeding. (A) Global vision. Note the low differentiated adenocarcinoma (less than 25% of glandular formation). (B) Amplification of the square from image A. Note the mitotic figures (MF), including atypical and signet ring cells (SRC), fibrotic beams separating the “nests” of tumor cells, pleomorphic cells with acidophilic cytoplasm (AC), and the vessel structure in the connective tissue (BV). [HE A, 40×; HE B, 400×].

Three (±1,09) days after xeno-transplantation tumor had 100 mm^3^. Untreated animals had tumors that presented as deep, fixed, and vascularized masses at the graft site. After incision, white tissue was observed, slightly lobulated and with a “fish-meat” aspect, which suggested a carcinoma tumor. Under microscopy, all tumors consisted of poorly differentiated adenocarcinoma with fibrotic beams separating the “nest” of desmoplastic tumor cells with frequent mitotic figures, including atypical forms and signet ring cells. Medium-and large-sized tumor cell clusters, acidophilus and ample cytoplasm, delicate chromatin nuclei, and evident nucleoli were also found. The connective tissue showed BV structures (Figure 1A and 1B).

### Treatment with Q3G or beva

**Figure 2:**
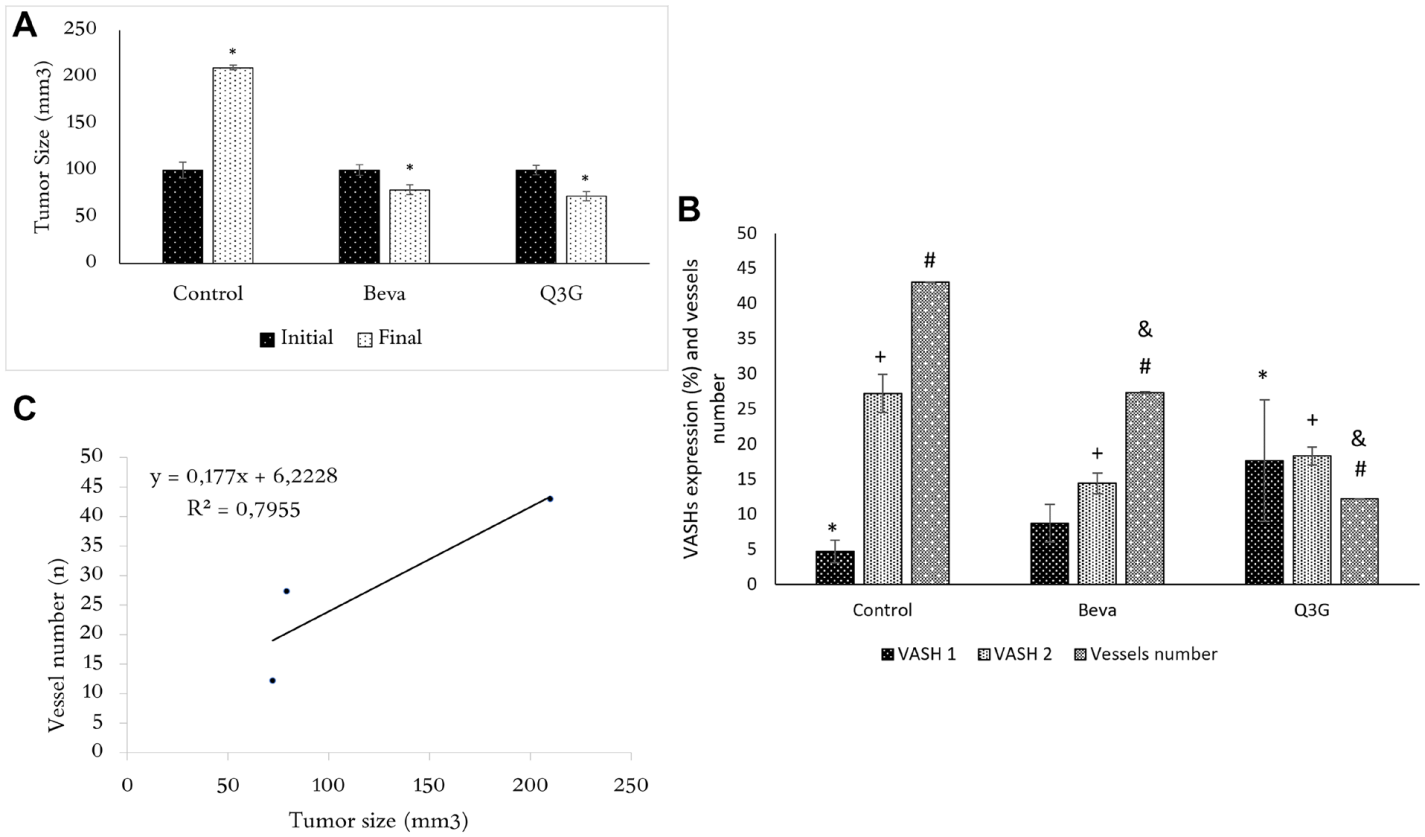
(**A**) Tumor size (mean ± SD) in untreated mice and beva- or Q3G-treated groups following inoculation of xenogeneic HT-29 tumor cells at the day of treatment (initial) and after 7 days (final). At the initial time (day 0), the tumor size is the same in untreated or Q3G- or beva-treated mice, whereas, after 7 days of Q3G- or beva-treatment, the tumors are smaller in both beva and Q3G groups compared to the untreated group. (*n* = 15, non-parametric ANOVA (Kruscal Wallis), ^*^ *p* < .05). (**B**) VASH1 and 2 CC expression and blood vessel content in untreated (control) and beva- or Q3G- treated groups. Expression of VASH1 is higher in Q3G-treated tumors compared to untreated mice. VASH2 is lower in Q3G-treated tumors compared to untreated and higher compared to beva-treated mice. Blood vessels amount is lower in Q3G-treated tumors compared to untreated and beva-treated mice. (*n* = 15, non-parametric ANOVA (Kruscal Wallis), ^*^ *p* < .05). (^*^compared to VASH1, ^+^compared toVASH2, ^#,&^compared to blood vessel number. = ^*,+,#,&^ = *p* < .05). (**C**) Relationship between tumor size and blood vessel content. Note the tumor size as an “angiogenesis-dependent” variable (*n* = 15, Spearman test, R^2^= adhesion to curve, rs= 0,79, *p* < .05).

Tumors were smaller and less vascularized in Q3G- and beva-treated mice than in untreated mice (Figure 2A). There were fewer BVs in the tumors of Q3G- and beva-treated animals than in untreated animals (Figure 2B). Treatment with Q3G lowered tumor BV content to a greater extent than beva treatment (Figure 2B). None suffer signals were found in animals under Q3G treatment. The weight from day 0 to euthanasia day mice was stable. Untreated mice showed weight from 19,58 ± 0,75 to 19,98 ± 1,20, Q3G-treated mice from 20,0 ± 0,04 to 20,22 ± 1,56 and beva-treated mice from 19,01 ± 2,10 to 21,23 ± 4,75 (*p* = 0.88).

VASH1 expression was increased by Q3G treatment, and VASH2 expression was lower in Q3G- and beva-treated CC compared to untreated CC, and was higher in Q3G-treated CC in comparison to beva treatment (Figure 2B). The paucity BVs in the Q3G- and beva-treated tumors corresponded with their smaller size (Figure 2C).

**Figure 3:**
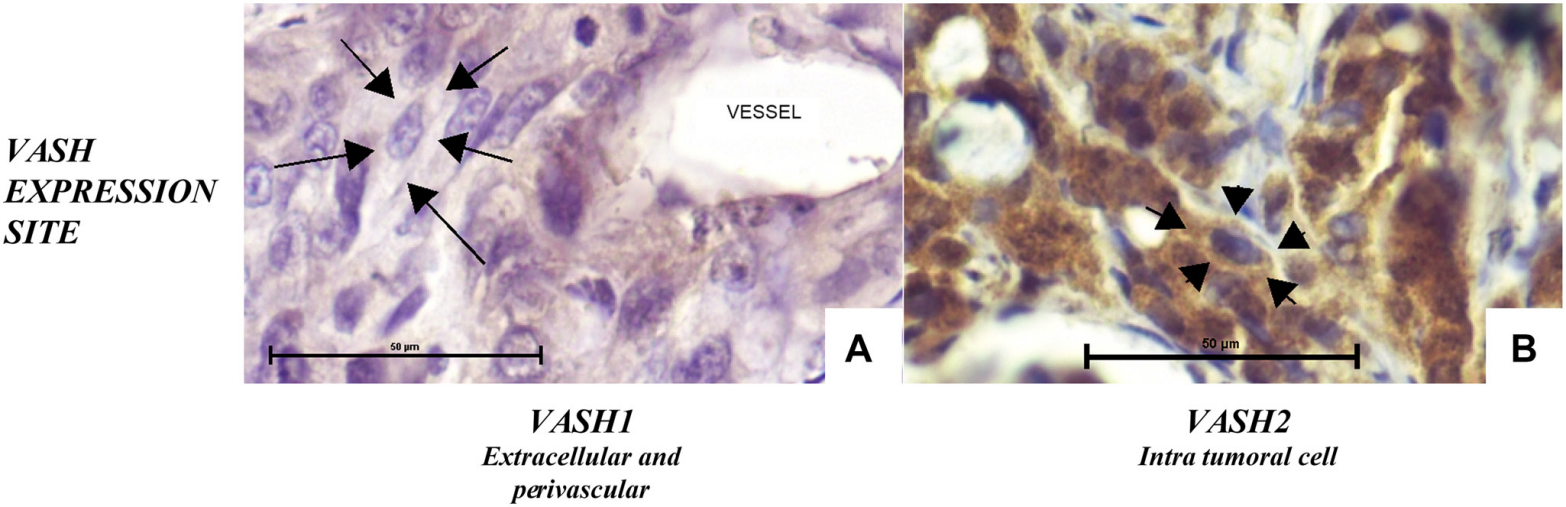
VASH1 (**A**) and VASH2 (**B**) expression in untreated CC. VASH1 was found in the extracellular matrix and endothelial cells, whereas VASH2 was found in the intracellular space. [Anti-VASH1 and anti-VASH2, 400×].

We found VASH1 or 2 expressions at distinct sites in CC (Figure 3A and 3B). VASH1 was found in the extracellular matrix close to BVs (Figure 3A) whereas VASH2 was found in the cytoplasm (Figure 3B).

**Figure 4:**
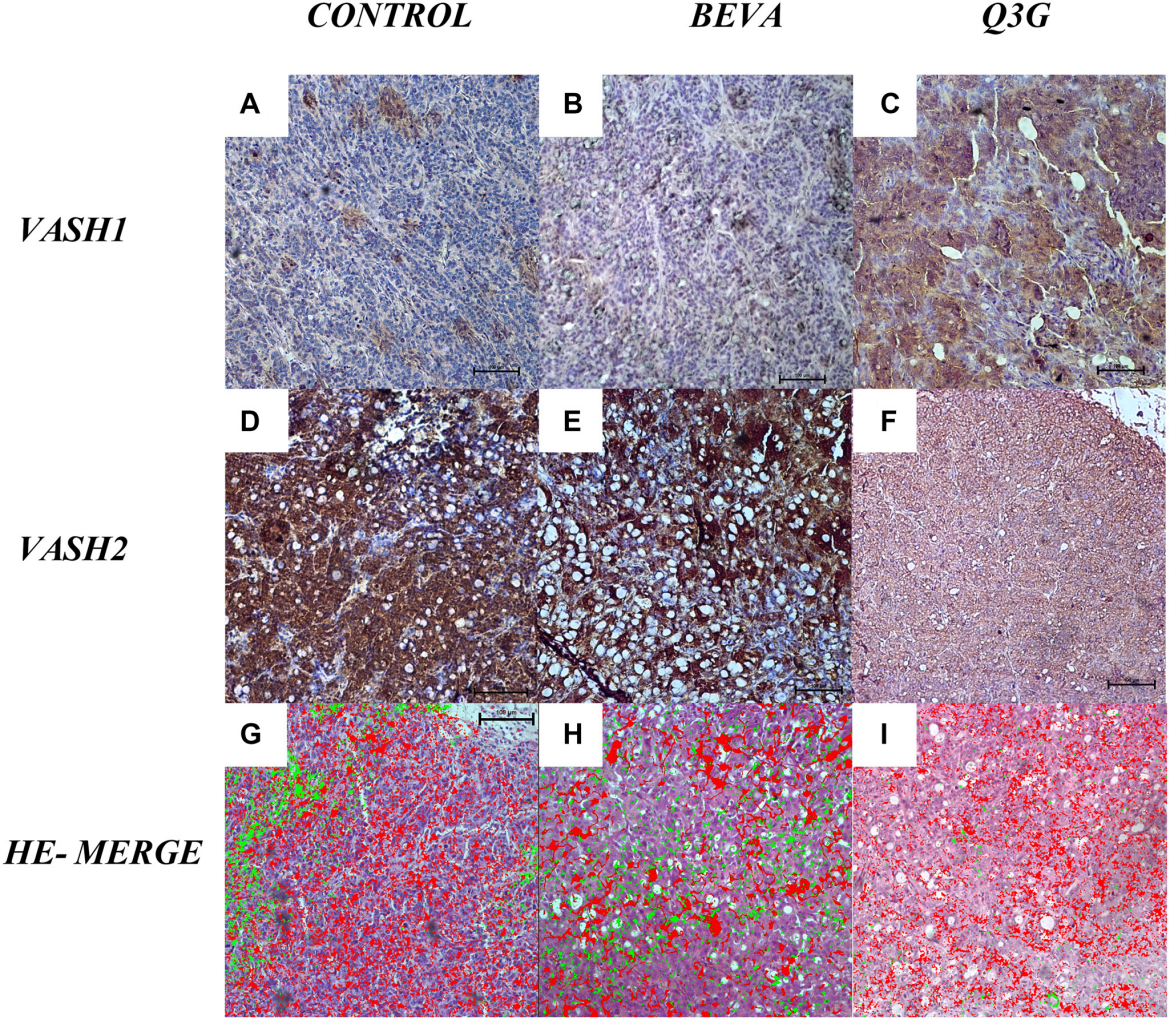
Tissue expression of VASHs (**A**–**F**) and VASHs-HE merged (**G**–**I**) in untreated (A, D, G), beva-treated (B, E, H), or Q3G-treated CC (C, F, I). VASH1 shows higher expression (C) and VASH2 lower expression (F) in Q3G-treated CC compared to the control [Anti-VASH1 and anti-VASH2, 100x]. HE-Merged (G, H, I) images show VASH1 (red- in the front proliferation zone) and VASH2 (green- in the termination zone) tissue expression in distinct sites in CC. VASH1 was detected in the perivascular matrix, and VASH-2 was intracellular. [HE, 40×].

In Q3G-treated tumors, VASH1 expression was higher (Figure 4C) than untreated (Figure 4A) and Beva treated (Figure 4B) tumors, and VASH2 expression was lower (Figure 4F) in comparison to untreated (Figure 4D) and Beva treated (Figure 4E) tumors. On merged micrographs, we found a lower expression of VASH1 in ECs proliferating at the sprouting front. In turn, high levels of VASH1 were detected in non-proliferating ECs in the angiogenesis termination zone. High levels of VASH2 were found at the sprouting front and lowered in the termination zone in the cytoplasm (Figure 4G–4I).

## DISCUSSION

Q3G and beva inhibited CC growth compared to untreated controls.Q3G showed CC inhibition in agreement with the known effects of flavonoids on several types of tumors and the antiproliferative effect of Q3G on CC cells *in vitro* [36–42, 55].

Q3G was safe to use. The clinical signs were used for the animal welfare refinement during animal experiments. The mice treated with Q3G showed no suffer signs. A 5% body weight loss is a strong empirical predictor of pathological findings as a non-invasive tool to monitor research animal welfare in toxicity testing [56]. Data showed stability of weight in mice treated with Q3G.

In addition to smaller tumor size, Q3G treatment lowered the amount of BVs within tumors, whereas larger untreated tumors had more BVs. CC treated with Q3G showed approximately 65% fewer BVs than untreated controls and 50% fewer BV than tumors treated with beva. The results confirm that cancer is an “angiogenesis-dependent” variable [15]. The growing tumors need an increase in blood supply to obtain sufficient oxygen and nutrients. One way to obtain this is by stimulating angiogenesis. As a result of tumor expansion, the production of angiogenic occurs. It led to an increase in the intravascular distance and placed tumor cells beyond the critical oxygen diffusion limits. During the initial phase of angiogenesis, the existing little BV become dilated, have increased permeability, and their endothelial cells show an increase in cytoplasmic organelles and a decrease of endothelial junctions. Subsequently, endothelial cells need to detach from and subsequently break down the basement membrane. They start migrating and proliferating to form new sprouts that enter growing tumor nests. Migrating endothelial cells align in loose cords and sprouts, and lumen formation occurs in the advancing sprout by vacuole formation and curving of the endothelial skeleton. When these sprouts become connected, blood flow will start [57]. These connected sprouts allow the invaded tumor nests to grow and will themselves develop blood vessels and new sprouts leading to expansion of the tumor BV and tumor growth [58]. Therefore, cancer (growth, progression, and spread) is an angiogenesis-dependent variable. Unprecedentedly, Q3G interfered with the vascularization of CC. In addition, Q3G-treated animals had higher VASH1 and lower VASH2 levels compared to untreated animals. The literature shows that inhibition of tumor growth occurs in response to impairment of angiogenesis by rutin [59, 60]. Rutin is a flavonoid from the same class of Q3G, which corroborates its antiangiogenic activity. Furthermore the literature reported that kaempferol interferes with VASH1 expression [36] and that engeletin regulates VASH2 [54], which is consistent with the regulation of both angiogenic factors by flavonoids.

VASH1 and 2 have opposing activities that are linked to their expression sites. Antiangiogenic agents inhibit VASH production by blocking and sequestering VEGFR, which leads to EC apoptosis and vessel number reduction [61]. However, VASH1 expression is induced by numerous stimuli [62], including VASH2, and not exclusively by VEGFR.

Untreated tumors showed VASH1 mainly close to vascular structures in the extracellular matrix, whereas VASH2 was found within tumor cells. VASH1 impairs EC proliferation, migration, survival, and vascular permeability, which leads to angiogenic remodeling by inhibiting angiogenic sprouting and vessel growth [63]. In untreated animals, we found low tissue expression of VASH1 and high VASH2. Q3G-treated animals showed higher tissue VASH1 expression localized in the extracellular matrix close to the EC proliferating zone in comparison to the control. In turn, VASH2 was found in the cytoplasm of tumor cells and was downregulated in comparison to cancer cells from control mice. The VASH1 was detected in the extracellular matrix (ECM) and EC but not in tumor cells. The spatial in ECM is crucial to ensure the proper assembly and maturation of new vascular structures by angiogenic signals. Consequently, the presence of VASH in ECM was expected. Distinct from VASH1, VASH2 was also found in the tumor cells. It is an exciting characteristic and suggests VASH2 as an inhibitor of angiogenic development mediated by the tumor cell. There are therapeutic challenges for delivering antiangiogenic, including controlling the microenvironmental distribution of their levels in tissue [64]. The VASH2 as a potential therapeutic strategy is less dependent on the ECM and molecular structure. It considers the target of the tumor cells, pointing to a therapeutic strategy not based on VASH1 and 2 molecular structures, but on their different expression sites. Although the evidence that human colon adenocarcinoma is angiogenesis-dependent, angiogenesis is not the only factor determining tumor growth, including genetic changes and biochemical pathways that play a crucial role. Tumor treatment with agents direct to only one target involved in tumor development and progression and spreading seems insufficient to induce complete tumor regression response and subsequent improvement of the disease-free and overall survival. Currently, distinct therapies should be maintained to interfere with different hallmarks of carcinogenesis, but the association with Q3G seems to be promising.

The distinct secondary pharmacology of the agents must be considered to understand Q3G activity. Angiogenic agents are segregated based on VDA or AIA effects and are organized based on their chemical structures. Many drugs, even those designed to act specifically on a target protein, bind unintended proteins and can exhibit off-target activity and thereby display dual mechanisms of action or pleiotropism. Most VDAs bind to tubulin or destabilize tubulin polymerization. Flavonoids such as FAA [33] and vadimezan [34] interfere with tubulin; thus, they are grouped as VDAs. We speculate that Q3G is a VDA mainly because of its interference with VASH expression, similar to kaempferol [36] which regulates VASH1 and Engeletin [54] that acts on VASH2.

VASH1 and 2 control tubulin detyrosination and thereby control the detyrosination status of polymerized microtubules. Tubulin detyrosination is implicated in many cell functions, such as cell division, and interferes with angiogenesis *in vivo* [65]. VASH1 increases tubulin levels, and VASH2 exerts its activity in microtubules. The tubulin carboxypeptidase activity of VASH1 inhibits angiogenesis by interfering with endocytosis and trafficking of receptors for pro-angiogenic factor. Recently, VASH1 was found to mediate tubulin detyrosination, a post-translational modification that allows the discrimination of mitotic errors that need to be corrected to prevent chromosomal instability, and is implicated in tumor evolution and metastasis [66, 67]. Additionally, VASH2 has microtubule functions and exhibits proangiogenic activity [67]. Considering the regulation of VASH1 and 2 and tubulin by Q3G, we suggest that Q3G has antiangiogenic activity in CC. Although the experimental design of this study proves that Q3G changes VASH1 and 2 expressions, and the number of vessels is correlated to VASHs expressions, the direct effect of VASHs expression under blood vessels deserves investigations after some VASH inhibition, like silencing or maybe using antibodies against VASHs.

Previous research has recognized the importance of the local tumor microenvironment in tumor progression and its role during carcinogenesis [14, 68]. VASH2 is expressed in CC cells and accelerates tumor angiogenesis and progression. Nevertheless, it is not expressed in most normal adult tissues [8], which suggests that it would offer more tumor-specific favorable outcomes for CC. VASH2 inhibition may be a useful therapeutic strategy for CC. The greater specificity of VASH2 than 1 in tumor cells suggests that VASH2 is a suitable target for blocking angiogenesis in CC.

In summary, we determined the antitumor activity of Q3G in a xenogeneic CC mouse model. Unprecedentedly, Q3G interfered with the amount of BVs within tumors and regulated tissue VASH1 and 2 expression in CC. Although the experimental design of this study proves that Q3G changes VASH1 and 2 expressions, and the number of vessels is correlated to VASHs expressions, the direct effect of VASHs expression under blood vessels deserves investigations after some kind of VASH inhibition, like silencing or maybe using antibodies against VASHs.

## MATERIALS AND METHODS

This study followed the principles outlined in the US Public Health Service Policy on Humane Care and Use of Laboratory Animals, and Guidelines for the Welfare of Animals in Experimental Neoplasia in strict accordance with the guidelines of the National Council of Animal Experimentation Control (CONCEA) and ARRIVE [69]. The Research Ethics Committee approved the protocol of Sao Francisco University (USF) under permit number 001.05.12.

The study involved 15 male Balb-c nude mice (6–8 weeks old, 20 g of weight) from Charles River Laboratory (Wilmington, DE, USA). The study was conducted at USF. Animals received water and standard chow *ad libitum*, except for 30 min before the Q3G treatment. The mice were maintained in a ventilated light-controlled rack animal housing system with controlled humidity and temperature and were exposed to 12 h light-dark cycles. We used environmental enrichment techniques and animal welfare inspection guide [70] to minimize stress and suffering in animals. Clinical signs such as piloerection, eyes half shut, slightly decreased motor activity and body weight loss were used to monitoring the animal welfare.

### Xenogeneic CC animal model

Human CC/HT-29 cells (BCRJ code: 0111) were characterized by their DNA Profile (Amelogenin: X; CSF1PO: 11,12; D13S317: 11,12; D16S539: 11,12; D5S818: 11,12; D7S820: 10; THO1: 6,9; TPOX: 8,9; vWA: 17,19). These cells express urokinase receptors but do not have detectable plasminogen activator activity. HT-29 cells were negative for CD4, but there was cell-surface expression of galactose ceramide. These cells were obtained directly from Banco de Células do Rio de Janeiro (BCRJ, Brazil). Cells were thawed and propagated in culture following the International Guidelines on Good Cell Culture Practice [71]. Briefly, the cells were cultured at 37°C in a humidified chamber with 5% CO_2_ using Dulbecco’s modified Eagle medium (Sigma Aldrich, Brazil) supplemented with 100 mM sodium pyruvate (Gibco), 10% fetal bovine serum (Gibco, Thermo Fisher Scientific, Brazil), and 1% antibiotic (penicillin and streptomycin, Gibco, Thermo Fisher Scientific, Brazil). HT-29 cells were detached from the culture plate following incubation with 3 mL Trypsin-EDTA 0.25% (Gibco, Thermo Fisher Scientific, Brazil) for 3 min for transfer or harvest. The culture medium was changed every 24 h. Cell viability was evaluated using trypan blue staining. HT-29 cells (4 × 10^6^) were suspended in 40 μL of normal saline and directly seeded into the subcutaneous tissue of the back left flank of athymic mice through a percutaneous puncture (27-gauge hypodermic needle) using a 1 mL syringe [68, 72, 73].

After xeno-transplantation, tumor growth was checked daily using metal calipers. The formula “Volume = L × S × S/2”, where “S” is the minor diameter measured and “L” is the largest diameter measured, was used to determine the CC volume [68, 72, 74].

### Groups

The animals were randomly by lottery and distributed into three groups: untreated (negative control, *n* = 4), Q3G-treated (test, *n* = 6), or beva-treated (positive control, *n* = 5).

Treatment was initiated when tumor volumes reached 100 mm^3^. Untreated animals received 0,5 ml of vehicle (saline, 0.9% sodium chloride solution) treatment; Q3G-treated animals received 0,5 ml of Q3G (Quercetin-3-Beta-D-Glucoside, #17777793; Sigma Aldrich Brasil^®^) by gavage at the maximum non-lethal dose (0.017 mg/g body weight) over a week. The lethal dose of Q3G was determined as previously described by Chinedu et al., 2013 [75]. Beva-treated animals received beva (Roche, Brazil^®^) (25 mg/mL) by intraperitoneal injection (0,5 mL) at a single dose of 5 mg/kg body weight, based on currently recommended dosage for initial therapy in metastatic colorectal cancer [76]. Seven days after the treatment (Q3G or beva), all animals were euthanized by administration of parenteral anesthetic drugs.

### Histological analyses

The excised CC tissue was fixed in 10% formaldehyde solution, embedded in a paraffin block, and longitudinally sectioned. The slides of all tumors were named without group identification. Every slide was analyzed in three fields by three researchers. The data assumed the average among them. The slides (4 μm) were stained with hematoxylin and eosin (HE). The presence of CC, degree of differentiation, and microvascular quantification were determined. All identified vascular structures throughout the tumor surface were counted according to the International Consensus of Evaluation of Angiogenesis Quantification in Solid Human Tumors [77, 78]. Microvascular quantification was performed using computerized image processing software (NIS for Windows) [79, 80].

### Immunohistochemical analysis

Immunohistochemistry was performed using the avidin-biotin-peroxidase technique with an anti-VASH1 antibody (HPA000653-100UL, Lot A06367; Sigma-Aldrich Corporation^®^, Saint Louis, MO, USA) or anti-VASH2 antibody (ab224723, Lot GR3199655-29) diluted at a 1:100 ratio in phosphate-buffered saline with 1% bovine serum albumin (Sigma-Aldrich). Positive and negative immunohistochemistry controls were used to ensure the quality of the measurements. Immunoexpression microphotography and HE merged images were prepared to verify VASH1 and 2 tissue sites. A specific image analysis program (NIS for Windows) that combines the numerical values of the points in the color histogram that made up the image allows the user to determine the immunostaining by a numerical value in each field analyzed. To quantify VASH1 and 2 proteins a camera attached to the optical microscope captured the selected images in each slide. After capture, the images were evaluated by NIS Program. The measurement was made in a three field representative of the sample [79, 80]. The average obtained after reading separate fields on the same slide was considered as the VASHs expressions (%).

### Statistical analysis

We performed a pilot study using nine animals (three for each group) to establish power and used a significance level of 0.90 that determined a minimum sample size of 12 animals (at least four for each group). The Statistical Package for the Social Sciences (SPSS) version 21.0 for Windows (IBM Corp., Armonk, NY, USA) was used for data analysis following descriptive statistics; measures of central tendency; normality test; non-parametric ANOVA (Kruskal Wallis) to compare size, BV content, VASH1 and 2 tissue expression; and Spearman correlation associated with the tumor size and BV content. Data were expressed as average and standard deviation, and a p less than 5% (*p* < .05) was used to reject the null hypothesis.

### Data availability

Materials, data, and protocols should be made available upon request and within a reasonable amount of time from the corresponding author (depriolli@gmail.com) upon request.

## Abbreviations

BV: Blood vessels
EC: Endothelial cell
VEGF: Vascular endothelial growth factor
bFGF: basic fibroblast growth factor
TGF-β: Transforming growth factor-beta
FGF: Fibroblast growth factor
VEGFR2: Vascular endothelial growth factor receptor 2
FGFR1: Fibroblast growth factor receptor 1
VASH: Vasohibin
SVBP: Small vasohibin-binding protein
AIAs: Angiogenesis inhibiting agents
VDAs: Vascular disrupting agents
VTAs: Vascular targeting agents
beva: bevacizumab
CC: Colon cancer
IgG1: Immunoglobulin1
FAA: Flavone-8-acetic acid
DMXAA: Dimethylxanthenone-4-acetic acid
Q3G: Quercetin-3-O-β-d-glucopyranoside
CONCEA: National Council of Animal Experimentation Control
HE: Hematoxylin and eosin
SPSS: Statistical Package for the Social Sciences
